# APOBEC3B Promotes SARS-CoV-2 Through Activation of PKR/eIF2⍺ and AMPD2 Dysregulation

**DOI:** 10.3390/v17091176

**Published:** 2025-08-28

**Authors:** Benjamin Fixman, Lavanya Manjunath, Philip Sell, Shanshan Wang, Tamara Margaryan, Connor Qiu, Hanjing Yang, Rémi Buisson, Xiaojiang S. Chen

**Affiliations:** 1 Molecular and Computational Biology, Department of Biological Sciences, University of Southern, Los Angeles, CA 90089, USA; fixman@usc.edu (B.F.); wangshan@usc.edu (S.W.); tamaram2@usc.edu (T.M.); ccqiu@usc.edu (C.Q.); hanjingy@usc.edu (H.Y.); 2 Department of Biological Chemistry, School of Medicine, University of California Irvine, Irvine, CA 92697, USA; lmanjuna@uci.edu (L.M.); rbuisson@uci.edu (R.B.); 3 Department of Molecular Microbiology and Immunology, The Hastings and Wright Laboratories, Keck School of Medicine, University of Southern California, Los Angeles, CA 90089, USA; phil@metaba.us; 4 Center of Excellence in NanoBiophysics, Los Angeles, CA 90089, USA; 5 Norris Comprehensive Cancer Center, University of Southern California, Los Angeles, CA 90089, USA

**Keywords:** APOBEC3B (A3B), SARS-CoV-2 infectivity, severe COVID-19, PKR-mediated integrated stress response, Proviral role, Geneformer, AMPD2, immune regulatory networks

## Abstract

APOBEC3B (A3B) has been implicated in host–virus interactions, but its role in SARS-CoV-2 infection is unclear. Here, we demonstrate that A3B is overexpressed in bronchoalveolar lavage fluid (BALF) cells from severe COVID-19 patients compared to those with mild disease. A3B knockdown in Caco-2 cells significantly reduces SARS-CoV-2 infectivity, likely through attenuation of the PKR-mediated integrated stress response, a pathway proposed to promote SARS-CoV-2. Single-cell RNA sequencing (scRNA-seq) data suggest that BALF cells from severe COVID-19 patients exhibit a repressed state for cellular translation, potentially mediated by eIF2α phosphorylation. However, in A549-ACE2 cells, SARS-CoV-2 does not activate PKR, but A3B knockdown still reduces SARS-CoV-2 infectivity, suggesting an alternative mechanism of action in different cellular contexts. To further investigate A3B’s role in severe COVID-19, we employed Geneformer, a transformer-based machine learning model, which predicted that A3B knockout would perturb AMPD2 (adenosine monophosphate deaminase 2), a key enzyme in purine metabolism and immune regulation. We validated this prediction using bulk RNA-seq and clinical scRNA-seq data, confirming that AMPD2 expression is downregulated in severe COVID-19 but restored upon A3B knockdown. Together, these findings suggest that A3B plays a proviral role in SARS-CoV-2 infection by modulating translational control and immune regulatory networks, warranting further studies to elucidate the underlying mechanistic details.

## 1. Introduction

Apolipoprotein B mRNA editing enzyme, catalytic polypeptide 3B (APOBEC3B) is a member of the 11-protein APOBEC family of cytidine deaminases. All APOBEC family members share a conserved cytidine deaminase domain [1], characterized by the conserved His-X-Glu-X_23-28_-Pro-Cys-X_2-4_-Cys consensus sequence [2,3], and are believed to have evolved through a series of duplication events and subsequent diversifications [1]. The seven member APOBEC3 sub-family is clustered in tandem on chromosome 22q13.1 [1] and consists of both single-domain (APOBEC3A, APOBEC3C, and APOBEC3H) and double-domain (APOBEC3B, APOBEC3DE, APOBEC3F, and APOBEC3G) proteins [4]. While the C-terminal deaminase domain (CTD or CD2) catalyzes cytidine deamination, the N-terminal deaminase domain (NTD or CD1), though catalytically inactive, enhances substrate binding and deamination efficiency [1,5,6].

The APOBEC3 sub-family gained prominence in the early 2000s, when it was discovered that they can function as intrinsic antiviral factors against human immunodeficiency virus infection [7]. Initially, this anti-HIV function was primarily thought to result from the catalytic cytosine-to-uracil mutations on HIV cDNA [8], but it was later determined that APOBEC3s have both deamination-dependent and deamination-independent mechanisms for restricting retroviral infection [9]. Additionally, it soon became clear that, in addition to HIV, APOBEC3s played a role in restricting other retroviruses and para-retroviruses such as human T cell lymphotropic virus 1 [10,11], and hepatitis B virus [12,13]; single-stranded DNA viruses such as parvovirus [14,15]; and double-stranded DNA viruses such as human papillomavirus [16,17]. Herpes viruses such as Epstein–Barr virus (EBV) [18], Kaposi’s sarcoma herpes virus [19], cytomegalovirus [20,21], and herpes simplex virus [22] have all been shown to upregulate APOBEC expression. Moreover, several herpesviruses (such as EBV) use the viral-encoded ribonucleotide reductase (RNR) large subunit to bind to the A3B active site, leading to the inactivation of A3B deaminase activity and the relocalization from the nucleus to the cytoplasm [23].

While APOBEC3s are known for their antiviral roles, APOBEC3-mediated mutation of viral DNA in polyomavirus and HIV is reported to provide evolutionary fuel for the viruses, allowing them to escape immune detection in vivo [24,25]. Recently, cell culture experiments have shown that APOBEC3A-mediated mutations of viral RNA promote severe acute respiratory syndrome coronavirus-2 (SARS-CoV-2) infectivity and fitness [26]. However, to date, scarce studies have described a deamination-independent, proviral role for APOBEC proteins.

Recently, a study showed that APOBEC3B (A3B) uses a deaminase-independent antiviral mechanism to restrict Sendai virus (SeV) [27]. After SeV infection, A3B was shown to activate protein kinase R that phosphorylates eIF2⍺ to repress protein translation [28]. By doing so, A3B promotes a cellular antiviral pathway that downregulates total protein synthesis to reduce the expression of viral proteins [29]. Interestingly, previous studies have shown that several viruses, including SARS-CoV-2 [30,31,32], Zika, and Dengue viruses [33], are able to take advantage of non-canonical translation initiation pathways, allowing these viruses to avoid translation repression caused by PKR-induced eIF2⍺ phosphorylation.

In addition to PKR-mediated translational repression, SARS-CoV-2 has been shown to hijack host nucleotide biosynthesis and inflammatory response pathways to promote viral infectivity [34]. Qin et al. [34] showed that SARS-CoV-2 NSP9 promotes de novo purine synthesis, building upon a previous finding that SARS-CoV-2 infection dysregulates purine metabolism and is significantly associated with cytokine release in COVID-19 patients [35]. More specifically, they found that COVID-19 patients had significantly increased serum AMP levels and that these were positively correlated with cytokine release syndrome (CRS)-related cytokines IL-10 and IL-18, which displayed progressive increases from healthy controls to mild and severe patients [35]. From these studies, it appears that SARS-CoV-2 hijacks host metabolism to increase flux through the purine de novo biosynthesis pathway while altering purinergic signaling to increase pro-inflammatory cytokine release.

Geneformer [36,37] is a context-aware, attention-based deep learning model, representing a state-of-the-art tool in the burgeoning field of context-specific network biology. Leveraging a massive pretraining database of over 95 million single-cell RNA-sequencing (scRNA-Seq) transcriptomes across diverse cellular and pathological contexts [37], Geneformer predicts gene network dynamics under previously unseen conditions. The model can either be fine-tuned on new datasets for specialized predictive tasks or used “out of the box” for multiple analyses. One of its most powerful features is its in silico perturbation analysis, in which a gene’s expression can be up- or downregulated (in silico) within a specific experimental or clinical context. Using its pretraining knowledge, Geneformer predicts transcriptome-wide changes in gene embeddings, where larger shifts in cosine similarity indicate stronger regulatory effects. This function was previously used to predict genes whose perturbation could shift diseased cardiomyocytes toward a healthy state [36]. Subsequent CRISPR-mediated knockouts validated these predictions, demonstrating restoration of cardiomyocyte contractility and disease phenotype rescue [36].

In this study, we present evidence of a proviral role for endogenous A3B, demonstrating that it enhances SARS-CoV-2 infectivity in two different cell lines, proposing two distinct mechanisms for the proviral activity of A3B. Specifically, we show that (1) SARS-CoV-2 infection activates the PKR/eIF2⍺ pathway in Caco-2 cells, and this activation of the PKR/eIF2⍺ pathway is A3B-dependent, resulting in promoting viral replication. (2) Using Geneformer, we predict a potential mechanism in which A3B knockdown triggers a compensatory upregulation of adenosine monophosphate deaminase 2 (AMPD2), thereby possibly altering purinergic inflammatory signaling and purine nucleotide biosynthesis, reducing SARS-CoV-2 infectivity. These findings provide a potential paradigm shift in our understanding of A3B’s role in viral pathogenesis and suggest potential new therapeutic strategies targeting A3B-related translational, inflammatory, and metabolic pathways.

## 2. Results

### 2.1. A3B Is Overexpressed in Broncho-Alveolar Lavage Fluid from Patients with Severe SARS-CoV-2 Infection

**Figure 1:**
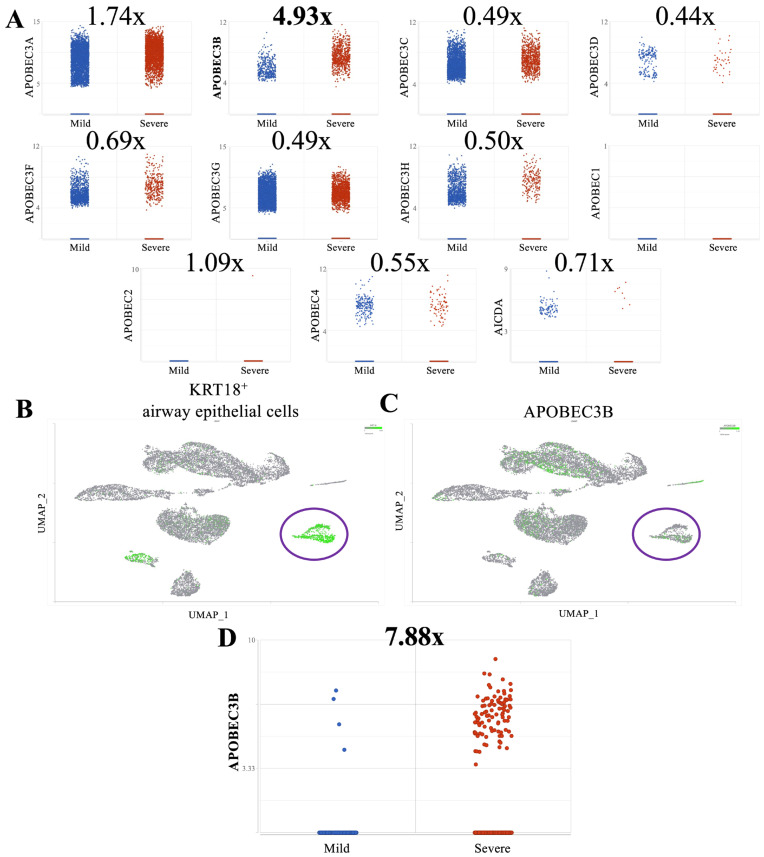
APOBEC3B is overexpressed in severe relative to mild COVID patient BALF: (**A**) Scatter plots showing log_2_ Normalized CPM+1 of APOBEC gene expression in mild (blue) and severe (red) COVID patients. Mean expression ratio (severe–mild) is shown above each plot. Cell counts were downsampled to c = 6318 (severe) to allow fair visual comparison with mild (c = 6316). FDR < 0.001 for all comparisons except APOBEC1 and APOBEC2. (**B**) UMAP plot showing distribution of cells from all samples and colored for epithelial airway marker KRT18 (green). Identified airway epithelial cells are circled in purple. (**C**) UMAP plot marked for A3B expression (green). Airway epithelial cells are circled. (**D**) Scatter plot showing log_2_ Normalized CPM+1 of APOBEC3B gene expression in airway epithelial cells, mild (blue) and severe (red) COVID patients.

To investigate differential expression of host restriction factors in SARS-CoV-2-infected patients [38], we analyzed single-cell RNA sequencing (scRNA-Seq) gene expression data from broncho-alveolar lavage fluid (BALF) of individuals with mild and severe COVID-19 (GSE145926) (https://doi.org/10.1038/s41591-020-0901-9, accessed on 1 September 2024) [38]. As expected, we observed a significant increase in A3A expression in patients with severe disease compared to mild (fold change = 1.74, FDR < 0.01; Figure 1A), corroborating previous findings that A3A may enhance SARS-CoV-2 infectivity [26]. We observed the strongest increase in expression of A3B (fold change = 4.93, FDR < 0.01; Figure 1A) in this analysis.

To determine whether elevated A3B expression in severe cases was driven by increased infiltration of immune cells, which are likely enriched in severe relative to mild patients, we subsetted airway epithelial cells using the known epithelial airway markers KRT18, KRT5, and TPPP3 [38] (Figure 1B, Supplementary Figure S1). Notably, differential expression analysis of A3B within this epithelial subset revealed an even greater upregulation in severe disease (fold change = 7.88, FDR < 0.01; Figure 1C,D). Furthermore, A3B was expressed in a higher percentage of total cells and airway epithelial cells in severe cases compared to mild cases (Supplementary Figure S2), which implies a potential role of A3B in COVID-19 pathogenesis.

### 2.2. A3B Knockdown Significantly Reduces SARS-CoV-2 Infectivity in Caco-2 Cells

To investigate the causal relationship between A3B expression and SARS-CoV-2 infectivity, we conducted a series of cell culture experiments using the SARS-CoV-2 USA-WA1/2020 strain and siRNA-mediated knockdown of endogenous A3B. We selected Caco-2 cells, a well-established model for SARS-CoV-2 infection [26,39,40], as our initial in vitro system.

**Figure 2:**
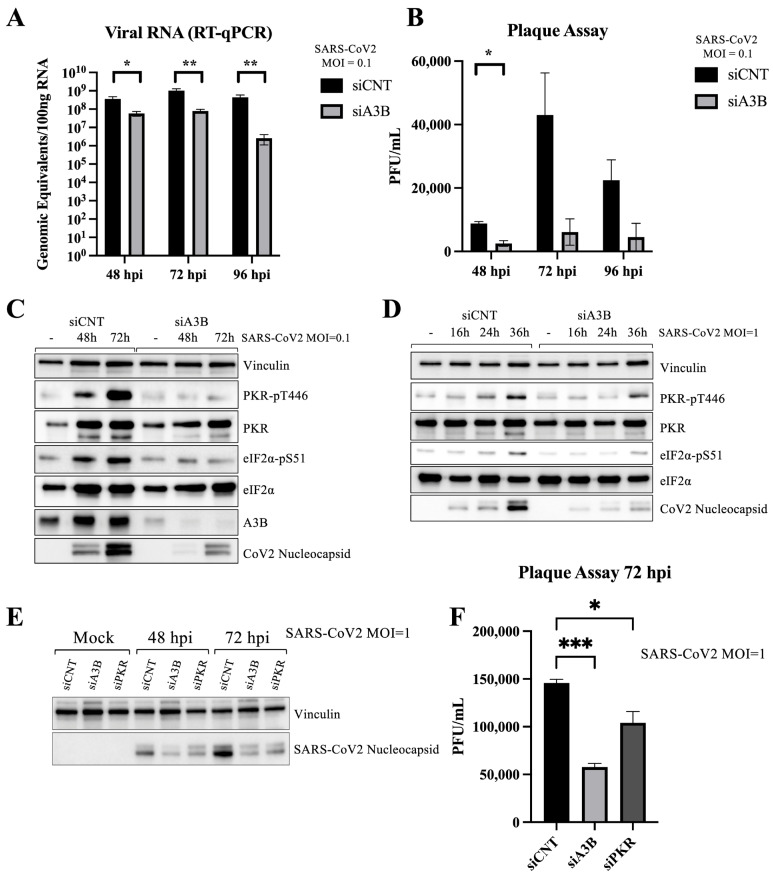
APOBEC3B knockdown reduces SARS-CoV-2 infectivity in Caco-2 through attenuation of p-PKR/p-eIF2⍺: (**A**) Genomic equivalents of SARS-CoV-2 at 2-, 3-, and 4 days post-infection with 8000 pfu. (**B**) Plaque-forming units/mL of media collected at 2-, 3-, and 4 days post-infection. (**C**) Western blot showing the levels of Vinculin, PKR, eIF2⍺, A3B, and SARS-CoV-2 nucleocapsid at 2- and 3 days post-infection (MOI = 0.1) in siCNT- and siA3B-treated cells. (**D**) Western blot showing the levels of Vinculin, GAPDH, PKR, eIF2⍺, and SARS-CoV-2 nucleocapsid at 16, 24, and 36 h post-infection (MOI = 1) in siCNT- and siA3B-treated cells. (**E**) Western blot showing the levels of Vinculin and intracellular SARS-CoV-2 nucleocapsid at 2- and 3 days post-infection in siCNT-, siA3B-, and siPKR-treated cells. (**F**) Plaque-forming units/mL of media collected at 3 days post-infection in siCNT-, siA3B-, and siPKR-treated cells. Bar graphs show mean ± SEM. Statistical significance was calculated by Student’s *t*-test: Statistical analyses were performed from data obtained from 3 experiments. * *p* < 0.05, ** *p* < 0.01, *** *p* < 0.001.

Cells were transfected with either siRNAs targeting A3B (siA3B) or non-targeting control siRNA (siCNT), followed by infection with 8000 pfu (MOI = 0.1). At 2-, 3-, and 4 days post-infection, we collected media supernatant and intracellular RNA for viral quantification. RT-qPCR analysis revealed a significant reduction in intracellular SARS-CoV-2 RNA following siA3B treatment (Figure 2A), while plaque assays revealed a decrease in secreted infectious virions in the supernatant at all three timepoints (Figure 2B), suggesting that A3B is required for SARS-CoV-2 replication in infected cells. Of note, we generated stable Caco-2 cell lines, overexpressing wild-type A3B (WT) or catalytically inactive mutant (E225A) A3B (IM). Upon infection with SARS-CoV-2, neither WT nor IM overexpression appeared to have a significant effect on SARS-CoV-2 infectivity (Supplementary Figure S3).

### 2.3. A3B Knockdown Reduces SARS-CoV-2 Infectivity in Caco-2 via Attenuation of PKR/eIF2⍺ Pathway

A recent study demonstrated that A3B promotes activation of the PKR/eIF2⍺ stress response pathway to dsRNA as a mechanism to restrict single-stranded RNA virus, known to activate PKR, such as SeV, poliovirus (PV), or Sindbis (SINV) infection [27]. To determine whether this pathway is involved in SARS-CoV-2 infection, we performed Western blot analysis 2 and 3 days post-infection in Caco-2 cells (Figure 2C). We found that SARS-CoV-2 infection increased phosphorylation (activation) of both PKR and eIF2⍺, indicating pathway activation. However, this activation was completely abolished upon A3B knockdown, suggesting that A3B is required for PKR-eIF2⍺ activation during infection. The activation of the PKR pathway was associated with an increase in intracellular SARS-CoV-2 nucleocapsid protein (Figure 2C). This effect persisted under higher viral load conditions (MOI = 1) and a shorter infection time course (16–36 h post-infection) (Figure 2D). Confirming our qPCR and plaque assay data, A3B knockdown showed a decrease in SARS-CoV-2 protein expression (Figure 2C–E).

Although PKR and eIF2⍺ are traditionally considered part of the host antiviral defense response [41], our observation suggests a proviral role for this pathway in SARS-CoV-2 infection. This aligns with recent work [30] showing that loss of eIF2A reduces SARS-CoV-2 replication, likely in part due to eIF2A’s essential role in supporting programmed-1 ribosomal frameshifting, which regulates the translation of SARS-CoV-2’s polycistronic RNAs. This recent study indicated that SARS-CoV-2 relied on non-canonical translation initiation pathways, relying on eIF2A, thus escaping the translation repression by eIF2⍺ phosphorylation that downregulates the host protein translation.

To determine whether PKR knockdown alone was sufficient to explain the reduction in SARS-CoV-2 observed with A3B knockdown, we performed PKR knockdown in Caco-2 cells, followed by Western blot analysis (Figure 2E) and plaque assay (Figure 2F) at 3 days post-infection. PKR knockdown led to a decreased intracellular viral nucleocapsid protein and reduced secretion of infectious virions.

### 2.4. Severe COVID-19 Patient BALF Cells Show Signs of p-PKR/p-eIF2⍺ Pathway Activation

**Figure 3:**
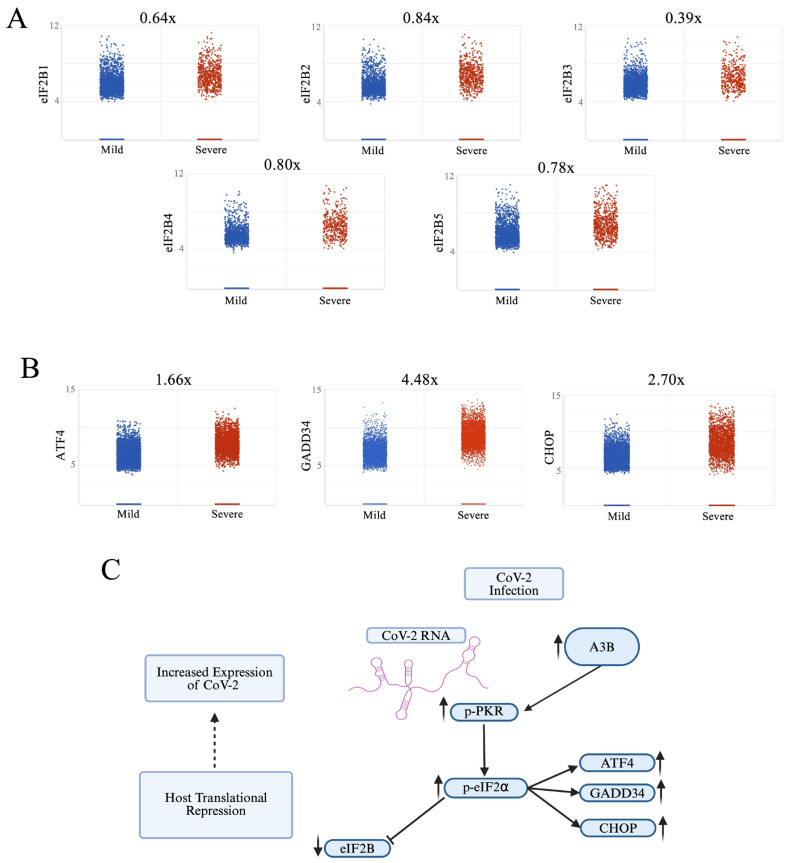
Severe COVID-19 patient BALF cells show signs of p-PKR/p-eIF2⍺ activation: (**A**) Increased eIF2⍺ phosphorylation (p-eIF2⍺) leading to translational repression leads to a decrease in expression of eIF2B, and (**B**) an upregulation of ATF4, GADD34, and CHOP expression. (**C**) Pathway overview in which SARS-CoV-2 infection induces an upregulation of APOBEC3B, driving an increase in p-PKR and p-eIF2⍺, leading to a decrease in eIF2B and increases in ATF4, GADD34, and CHOP.

It is challenging to demonstrate PKR/eIF2⍺ activation in RNA-sequencing data, as both proteins are regulated post-translationally through phosphorylation. Previous studies have indicated that activation of PKR/eIF2α does not increase their mRNA levels, despite increased levels of phosphorylated protein [30]. However, we used gene expression data of known markers indicative of eIF2⍺ phosphorylation (eIF2B, GADD34, and ATF4) [28] as surrogate measures for assessing increases in p-eIF2⍺ in vivo. In severe relative to mild COVID-19 patient BALF, we observed downregulation of all eIF2B isoforms (eIF2B1-5; Figure 3A) and an upregulation of GADD34, ATF4, and CHOP, all indicative of increases in p-eIF2⍺, suggesting that severe COVID-19 patient BALF cells are likely in a translationally repressed state, driven by eIF2⍺ phosphorylation. Similar results were previously shown by Bass et al. [42], who demonstrated that the downregulation of the eIF2⍺ pathway at the RNA level led to increased viral translation.

### 2.5. A3B Knockdown Reduces SARS-CoV-2 Infectivity in A549-ACE2 Independently of PKR Activation

**Figure 4:**
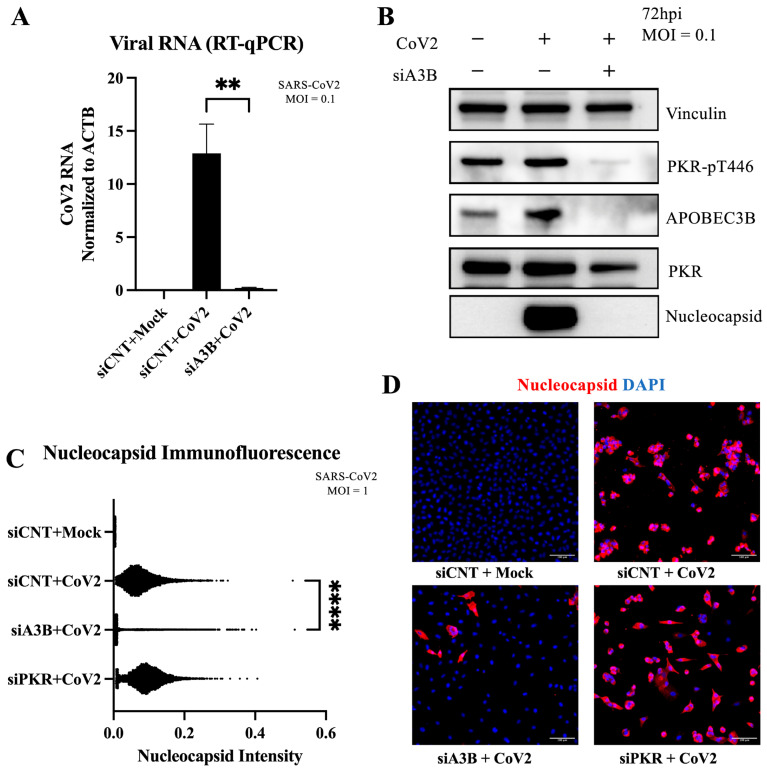
APOBEC3B knockdown reduces SARS-CoV-2 Infectivity in A549-ACE2 independent of p-PKR/p-eIF2⍺: (**A**) A549-ACE2 cells were infected at MOI = 0.1, and RNA was harvested 3 days post-infection for quantification by RT-qPCR. Bar graph shows mean ± SEM. (**B**) Western blot showing a decrease in intracellular SARS-CoV-2 nucleocapsid at 3 days post-infection with APOBEC3B knockdown, but no clear sign of PKR activation with infection. (**C**) Quantification of intracellular nucleocapsid intensity per cell as stained by immunofluorescence 3 days post-infection (MOI = 1), showing decreased nucleocapsid with APOBEC3B knockdown only. (**D**) Representative images showing nuclei (blue), CoV-2 nucleocapsid (red). Statistical significance was calculated by ANOVA followed by pairwise comparison with correction. Statistical analysis for qPCR was completed on 3 experiments. Statistical analysis on immunofluorescence was completed on 1 experiment. ** *p* < 0.01, **** *p* < 0.0001.

To determine whether this novel A3B-mediated proviral mechanism for CoV-2 infection was present in a second cell line, we infected A549-ACE2 and performed RT-qPCR to measure intracellular viral RNA at 3 days post-infection (MOI = 0.1 in Figure 4A, MOI = 1 in Supplementary Figure S4). As expected from previous observation in Caco-2 cells, we observed a roughly 65-fold decrease in viral RNA with siA3B treatment at MOI = 0.1. The reduction in viral RNA was roughly 7-fold at MOI = 1, possibly indicating that much higher initial viral concentration might overcome the restrictive effect of A3B knockdown. To determine if activation of PKR/eIF2⍺ was also driving this effect, we performed a Western blot analysis at 2 and 3 days post-infection. Interestingly, SARS-CoV-2 infection did not increase the levels of p-PKR with or without A3B knockdown (Figure 4B). However, A3B knockdown still resulted in a clear reduction in the viral nucleocapsid protein levels at 72 h (3 days) post-infection. Consistent with this observation is that PKR knockdown had no detectable effect on viral nucleocapsid protein levels (Figure 4C). These findings were further supported by immunofluorescence quantification, where A3B knockdown significantly reduced nucleocapsid intensity, but PKR knockdown did not (Figure 4D). Together, these results suggest that, unlike in Caco-2 cells, the decrease in SARS-CoV-2 infectivity upon A3B knockdown in A549 ACE2 cells is independent of p-PKR/p-eIF2⍺ pathway, indicating an alternative proviral mechanism distinct from that observed in Caco-2 cells.

### 2.6. Geneformer Predicts A3B Knockout Dysregulates AMPD2 in the Context of Severe SARS-CoV-2 Infection

Since A3B knockdown reduced SARS-CoV-2 infectivity in A549-ACE2 cells independently of its role in promoting the PKR/eIF2⍺ pathway, we sought to identify alternative mechanisms underlying this effect. To do so, we used Geneformer [36] to predict whether A3B knockdown alters gene network dynamics in the context of severe COVID-19, where A3B expression is high.

**Figure 5:**
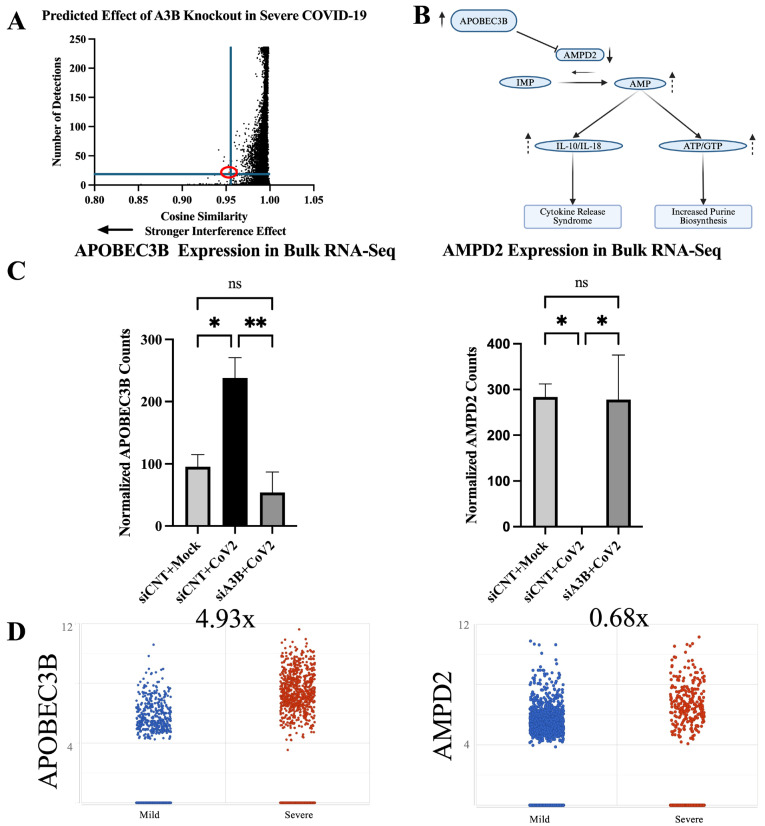
Geneformer predicts AMPD2 is dysregulated by APOBEC3B knockout in severe COVID-19 infection: (**A**) In silico deletion of APOBEC3B in airway epithelial cells of severe COVID patients revealed gene embedding predictions on 14,498 genes. AMPD2 is circled in red. (**B**) Pathway through which AMPD2 dysregulation could impact SARS-CoV-2 pathology. (**C**) Bulk RNA Seq shows that APOBEC3B expression is induced by SARS-CoV-2 infection and knocked down by siA3Bs (**left**). AMPD2 is knocked out with SARS-CoV-2 infection and has expression restored with APOBEC3B knockdown (**right**). (**D**) AMPD2 expression is reduced in severe relative to mild COVID-19 BALF (**right**); corresponding increase in APOBEC3B in severe relative to mild COVID (from Figure 1A, **left**). Bar graphs show mean ± SEM. Statistical significance was calculated by Student’s *t*-test, corrected for multiple comparisons. Statistical analyses were performed from data obtained from 3 experiments. * *p* < 0.05, ** *p* < 0.01.

We obtained the raw single-cell gene expression counts from airway epithelial cells of six severe COVID-19 patients (GSE145926) [38]. These counts were tokenized using Geneformer’s Transcriptome Tokenizer (*tokenize.data* function) and subsequently used as input in Geneformer’s in silico perturbation analysis. A3B (ENSG00000179750) expression was knocked out computationally using Geneformer’s *InSilicoPerturber* function, and gene embedding shifts were analyzed. A total of 14,498 gene embedding shifts were produced. Bulk RNA sequencing was performed on A549-ACE2 to validate the Geneformer prediction. Interestingly, adenosine monophosphate deaminase 2 (AMPD2), an enzyme that catalyzes the conversion of adenosine monophosphate to inosine monophosphate, exhibited the 5th largest shift in embedding of those genes with >20 detections (out of 9768 genes), indicating a strong interference effect (Figure 5A). It was indeed shown to have a statistically significant change in expression (FDR < 0.001) following A3B knockdown in the context of SARS-CoV-2 infection. This change was observed consistently across all three replicates and inversely correlated with A3B expression levels (r = −0.65; *p* = 0.06) when comparing mock infection, siCNT + SARS-CoV-2, and siA3B + SARS-CoV-2 (Figure 5C).

### 2.7. AMPD2 Is Downregulated in Severe COVID-19 Infection

To further investigate the clinical significance of AMPD2 in SARS-CoV-2 infection, we performed differential gene expression (DGE) using the data from GSE145926 (https://doi.org/10.1038/s41591-020-0901-9) [38]. The clinical data further corroborate the inverse relationship between A3B and AMPD2 expression in severe COVID-19. BALF samples from severely infected patients, which exhibited a 4.93-fold increase in A3B expression (Figure 1A, shown again in Figure 5D), demonstrated a corresponding 32% decrease (0.68-fold) in AMPD2 expression (Figure 5C), as well as a decreased proportion of cells expressing AMPD2 (Supplementary Figure S5). These results suggest that A3B upregulation in severe SARS-CoV-2 infection suppresses AMPD2 expression, potentially altering purine metabolism, energy homeostasis, and anti-inflammatory signaling in infected cells (Figure 5B).

## 3. Discussion

Previously, endogenous A3B has been mainly shown to play an antiviral role. However, a recent study by Shen et al. [43] showed that A3B was upregulated in peripheral blood mononuclear cells in severe and moderate COVID-19 patients, and that overexpression of exogenous A3B promoted SARS-CoV-2 pseudovirus infectivity. We report here that severe SARS-CoV-2 infection is associated with upregulated A3B expression in broncho-alveolar lavage fluid from the lung, and that knockdown of endogenous A3B significantly restricts wild-type SARS-CoV-2 infectivity. The mechanisms for the proviral activity of A3B for SARS-CoV-2 infection are cell-type-dependent, showing either PKR/eIF2⍺ pathway dependency in Caco-2 or independence in A549-ACE2. Our studies suggest two possible mechanisms: (1) In Caco-2, increased A3B drives the activation of the PKR/eIF2⍺ pathway to downregulate host protein translation, which is considered a classical antiviral response [27]. However, SARS-CoV-2 infection is not inhibited with the activation of this pathway; instead, SARS-CoV-2 exploits this pathway, likely to selectively enhance translation of its own viral proteins [30]. (2) In A549-ACE2, infection-mediated increased A3B did not activate PKR/eIF2⍺ pathway. Rather, it appears to suppress AMPD2 expression, possibly leading to the elevated purine nucleotide synthesis and altered anti-inflammatory purinergic signaling, contributing to cytokine release syndrome and worsening disease severity in COVID-19 patients.

Our finding that SARS-CoV-2 infection induces high levels of A3B expression, driving the activation of the p-PKR/p-eIF2⍺ stress response, which is hijacked by the virus for its own benefit, provides an example of novel APOBEC/virus interaction. Here, we demonstrate that A3B’s ability to drive p-PKR/p-eIF2⍺ [27] is actually co-opted by SARS-CoV-2 to promote viral infectivity and gene expression, likely through the use of SARS-CoV-2’s non-canonical translation initiation pathways [30,31]. Because new viral strains are emerging frequently, it is important to identify host proviral factors whose inhibition may be used as an antiviral pharmacological treatment strategy in multiple cases. This study identifies A3B as a potentially druggable target for treating SARS-CoV-2 and potentially other viruses, yet to be discovered, that rely on the same mechanism to promote infectivity.

Notably, we utilized Geneformer to generate a hypothesis in a scenario where the underlying mechanism remained elusive. Geneformer predicted that A3B knockout in the context of severe SARS-CoV-2 infection would dysregulate AMPD2 expression. Previous studies [34,35] support the role of AMPD2 and purine biosynthesis in SARS-CoV-2 infection and disease severity. To validate Geneformer’s prediction, we performed bulk RNA sequencing and differential gene expression analysis, comparing mock-infected, control-infected, and A3B knockdown-infected cells. We found that SARS-CoV-2 infection significantly reduced AMPD2 expression, supporting previous findings demonstrating an increased flux through the de novo purine biosynthesis pathway during SARS-CoV-2 infection [34]. Validating Geneformer’s prediction, A3B knockdown restored AMPD2 expression during SARS-CoV-2 infection.

AMPD2 is an anti-inflammatory gene previously shown to be downregulated during severe COVID-19 infection [44]. AMPD2 catalyzes the conversion of extracellular adenosine-monophosphate (AMP), a molecule implicated in inflammation, into inosine monophosphate (IMP) [45]. Given that elevated AMP levels correlate with increased inflammatory cytokines (IL-10 and IL-18) during COVID-19 infection [35], decreased AMPD2 may exacerbate disease pathology by allowing AMP accumulation [34], thereby driving excessive cytokine release. We propose that high A3B expression induced by SARS-CoV-2 infection may suppress AMPD2 expression, given A3B’s role in inflammation modulation. Specifically, elevated A3B expression occurs in response to viral infection and inflammatory signaling, potentially repressing AMPD2 to further increase AMP accumulation and subsequent cytokine release. This mechanism suggests a potential positive-feedback loop wherein SARS-CoV-2 infection elevates A3B via inflammatory signaling (such as JAK1/STAT3/NF-κB) [46,47,48], resulting in further amplification of the cytokine storm observed in severe COVID-19 cases. Such a feedback loop aligns with previous studies showing that the IL-6/JAK1/STAT3 pathway enhances A3B expression [49], which itself stabilizes IL-6 mRNA [50], intensifying inflammatory signaling pathways and functioning to help drive a pro-inflammatory response.

To explore a potential relationship between A3B and AMPD2, we examined scRNA sequencing data from BALF of severe and mild COVID-19 patients. We observed a trend toward elevated A3B expression and reduced AMPD2 expression in severe cases relative to mild infection. While this pattern does not establish causality, it raises the hypothesis of a possible inverse relationship between these two genes in the context of SARS-CoV-2 infection. By applying Geneformer, we generated preliminary insights into a candidate gene network that would have been challenging to identify using traditional next-generation sequencing analysis methods. However, the underlying mechanisms driving this putative relationship remain unclear and will require future experimental validation and mechanistic investigation. Additionally, Renner et al. [32] did not find a statistically significant change in AMPD2 expression at a shorter timepoint than ours (48 h vs. 72 h) post-infection.

Given our finding that SARS-CoV-2 infection induces A3B expression, which in turn promotes SARS-CoV-2 infectivity, we speculate on its potential implications for long-haul COVID-19 patients. A3B is a well-established source of DNA mutagenesis and is implicated in tumor evolution [51,52,53]. Prolonged A3B expression, particularly in the setting of chronic inflammation, can result in clustered hypermutation signatures known as *kataegis* [54,55], a mutational process associated with cancer progression. Because of the known risks of prolonged A3B activity, long COVID-19 patients should be monitored for potential immune cell and lung cancers displaying the A3B mutational signature [52,56]. Future studies should investigate whether persistent A3B upregulation in post-COVID-19 patients contributes to an increased risk of malignancy and whether targeting A3B activity could serve as a potential therapeutic intervention in severe COVID-19 patients.

## 4. Materials and Methods

### 4.1. Cell Culture

Cells were cultured at 37 °C in a 5% CO_2_ atmosphere using a ThermoScientific™ Forma Series II Water Jacket CO_2_ Incubator. Caco-2 (ATCC-#HTB-37) were maintained in Eagle’s Minimum Essential Media (EMEM) supplemented with 10% fetal bovine serum (FBS) and 1% penicillin–streptomycin (PS) (Gibco-#15140122 (Grand Island, NY, USA)). A549-ACE2 (BEI-#NR53821 (San Francisco, CA, USA)) cells were cultured in Dulbecco’s Modified Eagle’s Medium (DMEM) containing 4.5 g/L glucose, L-glutamine, and sodium pyruvate, supplemented with 10% FBS, 1% P/S, and 100 µg/µL blasticidin. Vero-e6-ACE2 cells were cultured in DMEM containing 4.5 g/L glucose, L-glutamine, and sodium pyruvate, supplemented with 10% FBS, 1% P/S, and 2.5 µg/mL puromycin [57].

### 4.2. RNA Interference

Small-interfering RNA (siRNA) was purchased from Thermo Fisher (Waltham, MA, USA) under the *Silencer-Select* product line (siRNA sequences and catalog numbers are available in Table 1. For each gene knockdown, two siRNAs were used per target gene to ensure effective knockdown. Cells were seeded into 6- or 12-well plates and transfected with 10 µM siRNA using Lipofectamine RNAiMax (Thermo Fisher, #13778150) in Opti-MEM™ and antibiotic-free media 2 days prior to infection. After 1 day, the media were replaced with standard culture media, and the cells were incubated until infection [57].

### 4.3. SARS-CoV-2 Virus Infections

SARS-CoV-2 USA-WA1/2020 was obtained from the University of Southern California (BSL3 Core) and was originally gifted from BEI Resources (NR-52281). All SARS-CoV-2 stock virus production, infection, and titration were performed as previously described [26], with some modifications. All work with SARS-CoV-2 was conducted within the BSL-3 Laboratories at USC. Vero E6-hACE2 cells were used for SARS-CoV-2 stock propagation. Cells were seeded at 3 × 10^6^ cells in a T75 flask for 24 h before infection with SARS-CoV-2 (plaque isolate USA-WA1/2020) at a multiplicity of infection (MOI) of 0.005. Virus-containing supernatant was collected approximately 72 h post-infection (hpi) [57].

Virus was titrated by plaque assay, as previously described [26]. Vero E6-hACE2 cells were seeded in 6-well plates, and once cultures reached 100% confluence, they were infected with serially diluted SARS-CoV-2 virus stock (500 µL per well). After 60 min of incubation on a gentle shaker in the cell-culture incubator, the medium was removed, and the cells were overlaid with a medium containing FBS-free DMEM and 1% low-melting-point agarose (Gibco #12100-046). After plaque formation around 72 hpi, cells were fixed with 4% paraformaldehyde (PFA) overnight at room temperature. Solid DMEM-agarose was removed, and cells were stained with 0.2% crystal violet. Plaques were counted on a lightbox to determine viral titers [57].

Caco-2, A549-ACE2, and Vero-e6-ACE2 cells were infected with SARS-CoV-2 in serum-free media (250 µL or 500 µL per well for 12- or 6-well plates, respectively) at 37 °C, 5%CO_2_ on a gentle shaker at the indicated MOI for 60 min to allow for viral adsorption. After adsorption, infection media were replaced with standard culture media, and infections were continued for the indicated duration of infection [57].

### 4.4. Reverse Transcription-Quantitative Polymerase Chain Reaction

Total RNA was extracted from SARS-CoV-2 or mock-infected cells using TRIzol™ Reagent (Thermo Fisher, #15596026). The purity and concentration of extracted RNA were measured with a Nanodrop spectrophotometer (Thermo #ND-ONEC-W). Briefly, 100 ng of total RNA was reverse-transcribed into complementary cDNA using Protoscript II (New England Biolabs, #M0368S (Ipswich, MA, USA)) in a 20 µL reaction containing 1 µL of 100µM gene-specific reverse primer or 2 µL of 100µM random hexamer primers (NEB #S1330S (Ipswich, MA, USA)), 4 µL 1x Protoscript II buffer, 1 µL of 10 mM dNTP, 1 µL of 0.1 M DTT, 8U RNase Inhibitor (NEB #M0314S), and 200U Protoscript II reverse transcriptase. Reverse transcription was performed at 42 °C for 60 min, followed by enzyme inactivation at 65 °C for 25 min.

Quantitative PCR (qPCR) was performed using SYBR Green-based detection in a Bio-Rad™ (Hercules, CA, USA) FGX Opus 96 Real-Time PCR System (Bio-Rad #12011319). Reactions were prepared in 20 µL volume per well containing 2 µL of cDNA, 1 µL of forward and reverse primers (10 µM each), 10 µL of 2x SYBR Green Master Mix (Bio-Rad #1725120), and 7 µL nuclease-free water. PCR cycling conditions were set as follows: (95C × 30 s, ((95C × 5 s, 60C × 30 s) × 39)). Gene expression levels were quantified using either standard curve quantification based on SARS-CoV-2 N gene standard (IDT #10006625 (Coralville, IA, USA)) or by relative quantification using the 2^−ΔΔCt^ method and normalized to β-actin expression. qPCR data were analyzed using Bio-Rad CFX Maestro v2.3. Primer sequences used in this study are listed in Table 2.

### 4.5. Western Blots

Samples were subjected to a standard SDS-PAGE protocol and were subsequently transferred onto a PVDF membrane (Sigma, #IPVH00010 (St. Louis, MO, USA)). The membrane was then blocked for 1 h at room temperature using a blocking buffer composed of TSB-T (1× TBS and 0.05% Tween-20, and 5% milk. The membrane was then incubated overnight at 4 °C in the blocking buffer containing the primary antibody. After incubation, the membrane was washed three times with TBS-T, then incubated for 1 h with a rabbit/mouse-HRP conjugated secondary antibody diluted in TBS-T and washed thrice with TBS-T again. Protein signals were detected using the SuperSignal West Dura Extended Duration Substrate (Thermo Scientific, #34075) and visualized using a ChemiDoc MP Imaging System (Bio-Rad) [57].

### 4.6. Immunofluorescence

Paraformaldehyde was used to fix the cells (3% paraformaldehyde and 2% sucrose in 1× PBS) for 20 min, followed by two washes with 1× PBS. Permeabilization was carried out with a buffer containing 1× PBS and 0.2% Triton X-100 for 5 min. Next, cells were washed twice with 1× PBS and blocked for 1 h in PBS-T (1× PBS and 0.05% Tween-20) supplemented with 2% BSA and 10% milk. Primary antibody incubation was performed at room temperature for 2 h in 1× PBS containing 2% BSA and 10% milk. Coverslips were washed thrice with PBS-T before being incubated for 1 h with the appropriate fluorophore-conjugated secondary antibodies (Alexa-488 or Cy3). Following three additional washes with PBS-T, cells were stained with DAPI (5 µg/mL, MilliporeSigma #D9542 (Burlington, MA, USA)), and coverslips were mounted using slow-fade mounting media (Thermo Fisher Scientific, #S36936). Imaging was conducted using a Leica DMi8 THUNDER microscope [57].

### 4.7. Bulk RNA Sequencing

A549-ACE2 were harvested 3 days post-infection using TRIzol™ Reagent (Thermo Fisher, #15596026). Frozen TRIzol lysates were shipped to a commercial sequencing facility for library preparation (poly-A selection) and RNA sequencing.

### 4.8. Bulk RNA Sequencing Analysis

All bulk RNA sequencing analyses were performed using Partek Flow™ (version 10) [58]. The analysis pipeline included base trimming from both ends (min QS = 30); filtering contaminants (rDNA, tRNA, and mtDNA) using Bowtie 2.2.5; adapter trimming using Cutadapt 1.12; alignment to hg38-CoV-2WA1 hybrid genome using STAR 2.7.8a; filtering low quality alignments (minimum quality = 20); quantifying to the annotation model (Partek E/M); noise reduction (maximum count > 10); count normalization using median ratio; and differential gene expression (DGE) analysis using DeSeq2 (version 1.16.1).

### 4.9. Single-Cell RNA Sequencing Analysis and Cell Type Identification

All single-cell RNA-sequencing data analyses were performed using Partek Flow™ (version 10). The analysis pipeline included filtering counts (<10% mitochondrial counts, <20% ribosomal counts, retaining 58368/108230 cells); noise reduction (exclude genes whose counts = 0 in 100% of cells); and count normalization (counts-per-million (CPM) +1 Log2 transformation). For DGE analysis, severe COVID-19 samples were then downsampled to 1053 cells/sample to allow fair comparison with mild COVID-19 samples; DGE analysis was completed using *GSA* and the Hurdle model.

To identify cell types, we performed the following steps after normalization: principal component analysis (PCA) using the top 3000 most variable features, followed by graph-based clustering; UMAP was then run using the top 10 PCs. Epithelial airway cells were defined by expression of *KRT18, KRT5*, and *TPPP3*.

### 4.10. Geneformer

Geneformer [36,37] was executed on Google Colab using an NVIDIA A100 GPU for transcriptome tokenization, in silico deletion, and statistical analysis. The code used to run Geneformer will be made publicly available on the first author’s GitHub repository (https://github.com/bfixman, accessed on 1 September 2024) upon publication.

### 4.11. Antibodies

The antibodies used in this study are listed in Table 3.

### 4.12. Statistical Analysis and Plotting

All data visualization was performed with Partek Flow™ (version 10) or GraphPad Prism™ (v10.4.0, Macintosh). Statistical significance was assessed by ANOVA followed by pairwise comparison with Student’s *t*-test with correction for multiple comparisons; scRNA-seq differential expression analysis was conducted using Gene-Specific-Analysis in Partek, while bulk RNA-seq differential expression analysis was conducted using DeSeq2 (version 1.16.1). For immunofluorescence image quantification, CellProfiler™ (Broad Institute (Cambridge, MA, USA), version 4.2.8) was used for automated image analysis and fluorescence intensity measurements. FIJI™ (version 2.14.0/1.54f) was used to overlay multiple channels for the representative images.

## Figures and Tables

**Table 1:** siRNA sequences.

siRNA	Sequence	Company	Catalog Number
Control		Thermo Fisher Scientific	4390843
APOBEC3B	CCUCAGUACCACGCAGAAATT	Thermo Fisher Scientific	s18411
APOBEC3B	GAGAUUCUCAGAUACCUGATT	Thermo Fisher Scientific	s18412
PKR	GGUGAAGGUAGAUCAAAGATT	Thermo Fisher Scientific	s11187
PKR	GACGGAAAGACUUACGUUATT	Thermo Fisher Scientific	s11185

**Table 2:** Primer sequences.

Primer	Sequence	Company
Actin—Forward	CTGGCACCCAGCACAATG	IDT DNA
Actin—Reverse	GCCGATCCACACGGAGTACT	IDT DNA
CoV-2 N1—Forward	GGACCCCAAAATCAGCGAAAT	IDT DNA
CoV-2 N1—Reverse	TTCTGGTTACTGCCAGTTGAATCTG	IDT DNA

**Table 3:** Antibodies.

Antibody	Isotype	Company	Catalog Number
SARS-CoV-2 Nucleocapsid	Rabbit monoclonal	Cell Signaling Technology (Danvers, MA, USA)	86326
GAPDH	Rabbit polyclonal	EMD Millipore (Burlington, MA, USA)	ABS16
Vinculin	Mouse monoclonal	Sigma (St. Louis, MO, USA)	V9264
PKR	Mouse monoclonal	BD Biosciences (San Jose, CA, USA)	610764
PKR-pT446	Rabbit monoclonal	Abcam (Cambridge, UK)	ab32036
eIF2⍺	Rabbit monoclonal	Cell Signaling Technology (Danvers, MA, USA)	5324T
eIF2⍺-pS51	Rabbit monoclonal	Abcam (Cambridge, UK)	32157
A3B	Rabbit monoclonal	Abcam (Cambridge, UK)	184990

## Data Availability

Data is contained within the article or supplementary material: The original contributions presented in this study are included in the article/supplementary material. Further inquiries can be directed to the corresponding author.

## Supplementary Materials

The following supporting information can be downloaded at: https://www.mdpi.com/article/10.3390/v17091176/s1, Supplementary 1: UMAP plot showing distribution of cells from all samples and colored for epithelial airway marker KRT5 (left) and TPPP3 (right). Identified airway epithelial cells are circled in purple; Supplementary 2: Proportion of total and airway epithelial cells expressing APOBECs from mild and severe COVID-19 patients; Supplementary 3: Genomic equivalents of SARS-CoV-2 at 4 days post-infection (MOI = 0.1) in A3B knockdown (siA3B), control knockdown (siCNT), A3B Inactive Mutant (E225A) overexpression (A3B IM), and A3B Wild-Type overexpression (A3B WT). *** *p* < 0.001; Supplementary 4: A549-ACE2 cells were infected at MOI = 1 and RNA was harvested 3 days post-infection for quantification by RT-qPCR. Bar graph shows mean ± SEM; Supplementary 5: Proportion of total and airway epithelial cells expressing AMPD2 from mild and severe COVID-19 patients.
