# Supplementary material for: APOBEC3B Promotes SARS-CoV-2 Through Activation of PKR/eIF2⍺ and AMPD2 Dysregulation
Source: Viruses. 2025 Aug 28;17(9):1176. doi: 10.3390/v17091176 (PMC12474056; doi:10.3390/v17091176)
Supplement: Supplementary file 1 [file viruses-17-01176-s001.zip › viruses-3801996-supplementary.pdf]

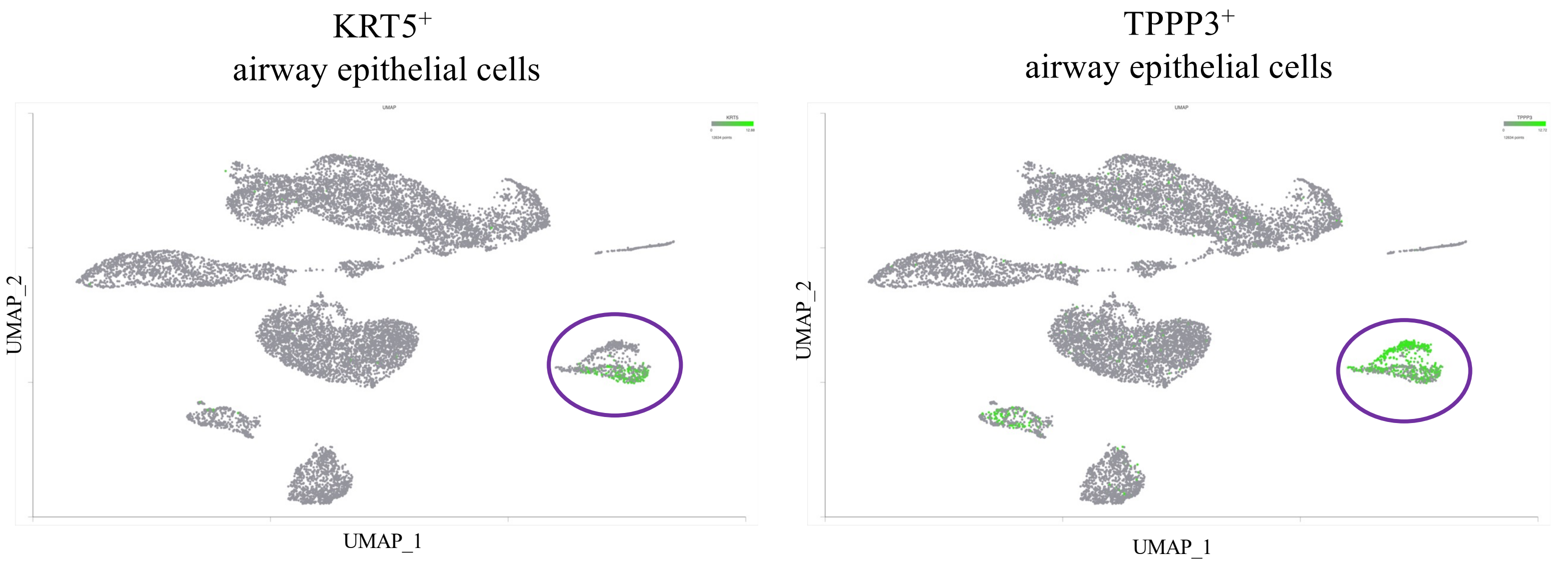

Supplementary Figure S1: UMAP plot showing distribution of cells from all samples and colored for epithelial airway marker KRT5 (left) and TPPP3 (right). Identified airway epithelial cells are circled in purple.

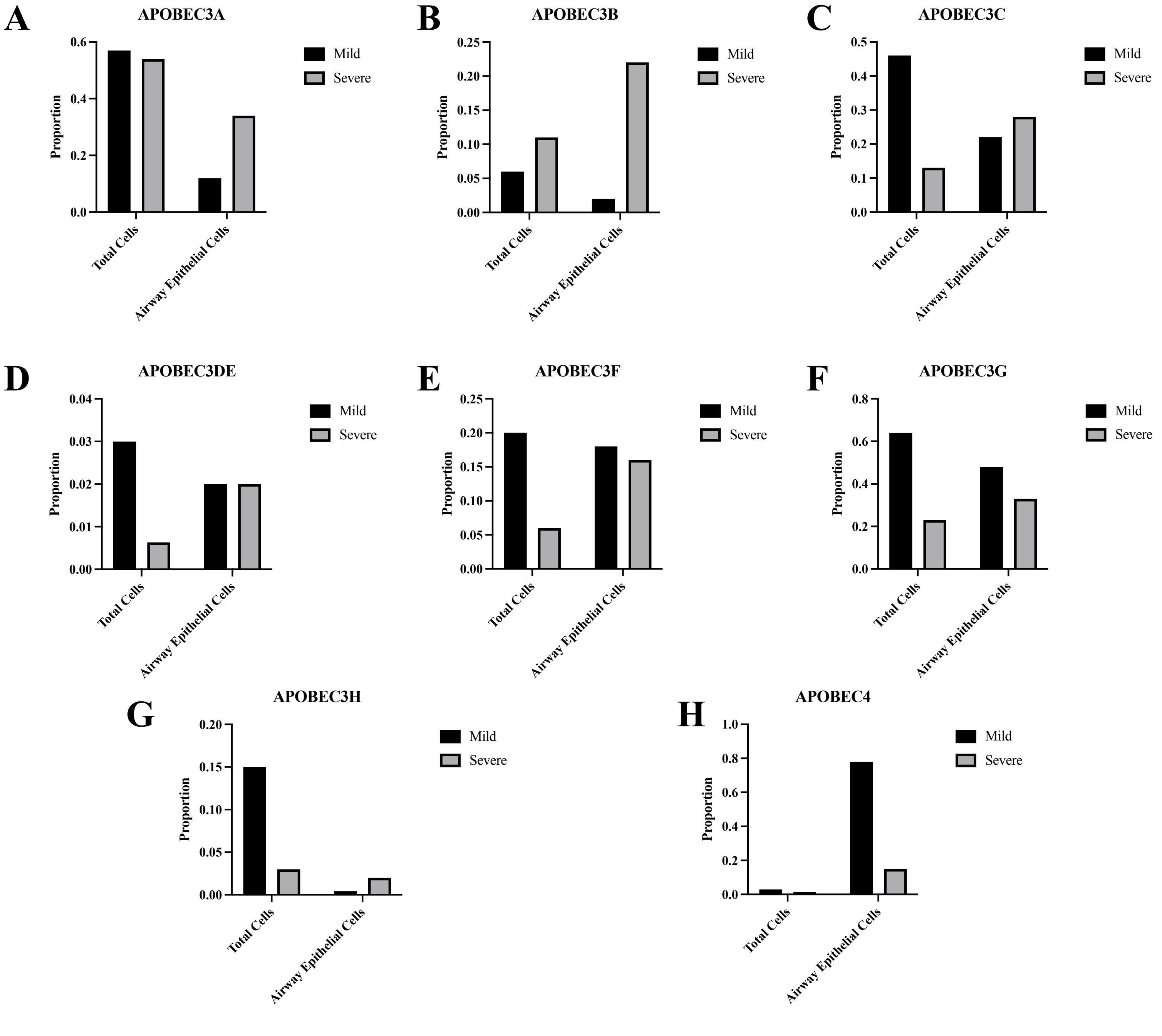

Supplementary Figure S2: (A-H) Proportion of total and airway epithelial cells expressing APOBECs from mild and severe COVID-19 patients.

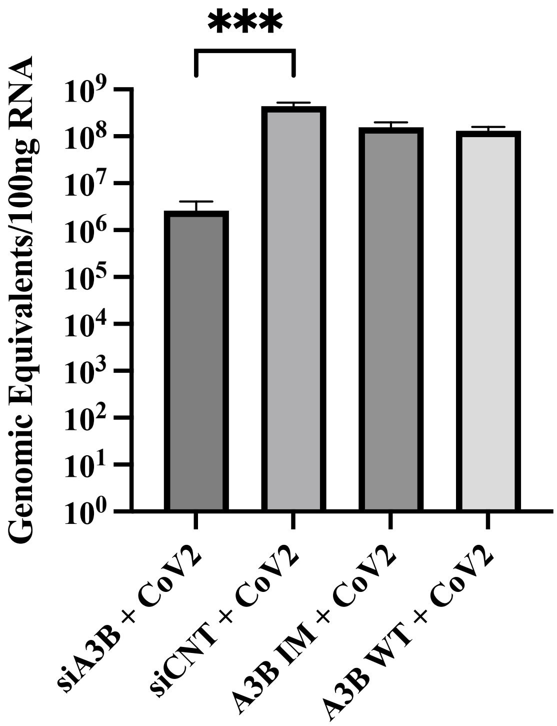

Supplementary Figure S3: Genomic equivalents of SARS-CoV-2 at 4 days post-infection (MOI=0.1) in A3B knockdown (siA3B), control knockdown (siCNT), A3B Inactive Mutant (E225A) overexpression (A3B IM), and A3B Wild-Type overexpression (A3B WT). *** $p < 0.001$.

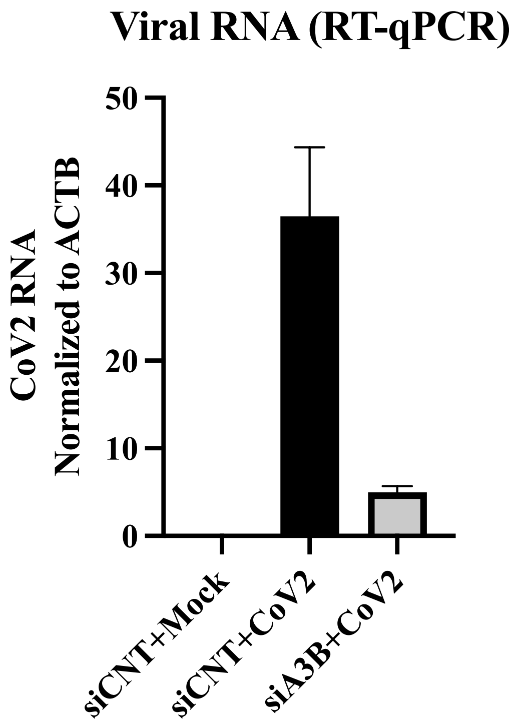

Supplementary Figure S4: A549-ACE2 cells were infected at MOI=1 and RNA was harvested 3 days post-infection for quantification by RT-qPCR. Bar graph shows mean ± SEM.

Supplementary Materials

APOBEC3B Promotes SARS-CoV-2 Through Activation of PKR/eIF2α and AMPD2 Dysregulation

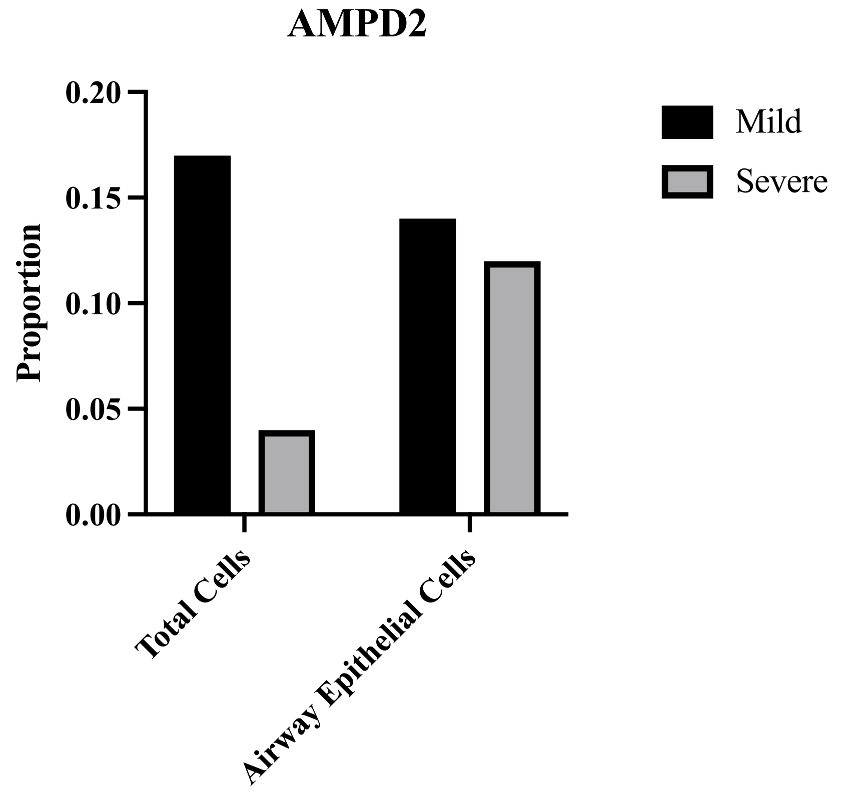

Supplementary Figure S5: Proportion of total and airway epithelial cells expressing AMPD2 from mild and severe COVID-19 patients.
